# Periscope of Dermatology

**Published:** 1895-08-31

**Authors:** 


### Periscope of Dermatology.

Hutchinson[38] records a case of mysosis fungoides in which the whole surface of the body, with the exception of the genitals, was almost uniformly involved. No definite tumours, however, were present. The patient steadily got worse in spite of treatment, but, unfortunately, no post-mortem examination was made.

Roberts[39] records another case of granuloma fungoides occurring in a woman forty-five years of age. Four or five years before, she had suffered from pruritus, but during the last year the entire cutaneous surface had become affected with tumours. These tumours were very numerous on the chest and had grown rapidly. Just prior to death there was rapid emaciation and shrinking of the tumours. Green[40] records the case of a man, aged sixty-one years, who suffered from a peculiar hypertrophy of the skin and subcutaneous tissue on the left side of the face, causing it to hang in folds. The patient, who was of dull intellect, stated that his face had always been similarly affected, and that the folds had formerly been more marked. There were also numerous warty growths on the trunk and subcutaneous tumours on the limbs. Besides these skin affections he was suffering from endosteal sarcoma of the left forearm.

**Therapeutics.**—Cantrell[41] reports on the use of losophan, and finds that it is entirely inefficacious in almost every disease of the skin. He therefore considers it a waste of time to make use of it in treating diseases of the skin.

Annequin[42] recommends a paste of calcium sulphydrate as a depilatory. The next best depilatory is the protosulphide of barium, which is also applied as a paste.

**Thyroid Feeding.**—Scatchard[43] reports a case of pityriasis rubra, which began to show signs of improvement within twelve days of the commencement of treatment by thyroid feeding, although the general health suffered considerably from the treatment. The patient became emaciated, depressed, and anæmic, and had dyspnœa. These symptoms, however, passed off on the stoppage of the thyroid feeding.

Nobbs[44] refers to a case of ichthyosis in a man aged forty-six years, who was also suffering from general paralysis. The skin affection was considerably benefited by thyroid feeding, and his general health improved at the same time, but the paralysis pursued its usual progressive course.

Preece[45] describes a case of psoriasis, of many years standing, which he treated with thyroid gland. The patient was a woman, aged 26, who had suffered from the eruption for as long as she could remember; after some relapses she is now apparently cured.

Jamieson[46] reviews the work of Bramwell and Abraham on thyroid fe ding, and describes the unpleasant symptoms to which this treatment sometimes gives rise.

**Miscellaneous.**—Abraham[47] reports the case of a boy, aged six years, suffering from a xerodermic condition of the skin of the face, limbs, and trunk, together with much pityriasis. He considered the case to be an example of Divergie's pityriasis rubra pilaris.

Madden[48] recommends the application of a concentrated solution of methylene blue in pruritus vualvæ. This should be applied after thoroughly aseptisizing the parts with a solution of bichloride of mercury.

Marcano[49] reports a case of leprosy, in a boy aged two years, in which a primary nodule had been diagnosed by bacteriological examination. This was freely excised, and there had been no further signs of the disease eight months after the operation.

Phillips[50] considers that the greater prevalence of baldness among men is due to the practice of wearing the hair close cut, since in this way we remove the gentle traction on the bulb and hair follicles which the natural weight of the hair exercises, and which constitutes the essential and natural stimulus necessary to secure the vascular supply to the hair-producing structures. Under such circumstances, more or less atrophy is the inexorable result.

Jamieson,[51] in a clinical lecture, on diseases of the skin, states that the treatment of skin affections is to be carried out exactly on the same principles as that of diseases of other parts. Rest is of primary importance; rest, together with the removal of all causes of irritation, then soothing treatment, and only cautious, very cautious stimulation.

[38] Brit. Journ. of Derm., March, 1895, p. 69. [39] Ditto. May, 1895, p. 143. [40] Lancet, April 20. 1895. p. 986. [41] Ther. Gaz., April 15, 1895, p. 221. [42] Ditto, Feb. 11, 1895. p. 118. [43] Brit. Med. Journ.. March 30, 1894, p. 695. [44] Ditto. ditto, p. 696. [45] Ditto. ditto, p. 697. [46] Ed. Med. Journ., May, 1895, p. 1,039. [47] Med. Week. April 12, 1825. [48] Les Nouv Remed., Jan. 8, 1895. [49] Med. Week. Feb. 15, 1895. [50] Birmingham Med. Rev., April, 1895, p. 217. [51] Clin. Journ., May 29, 1885, p 72.

